# Supplementary material for: Machine-Learning Classification Suggests That Many Alphaproteobacterial Prophages May Instead Be Gene Transfer Agents
Source: Genome Biol Evol. 2019 Sep 27;11(10):2941–53. doi: 10.1093/gbe/evz206 (PMC6821227; doi:10.1093/gbe/evz206)
Supplement: evz206_Supplementary_Data [file evz206_supplementary_data.zip › Supplementary_Figures.pdf]

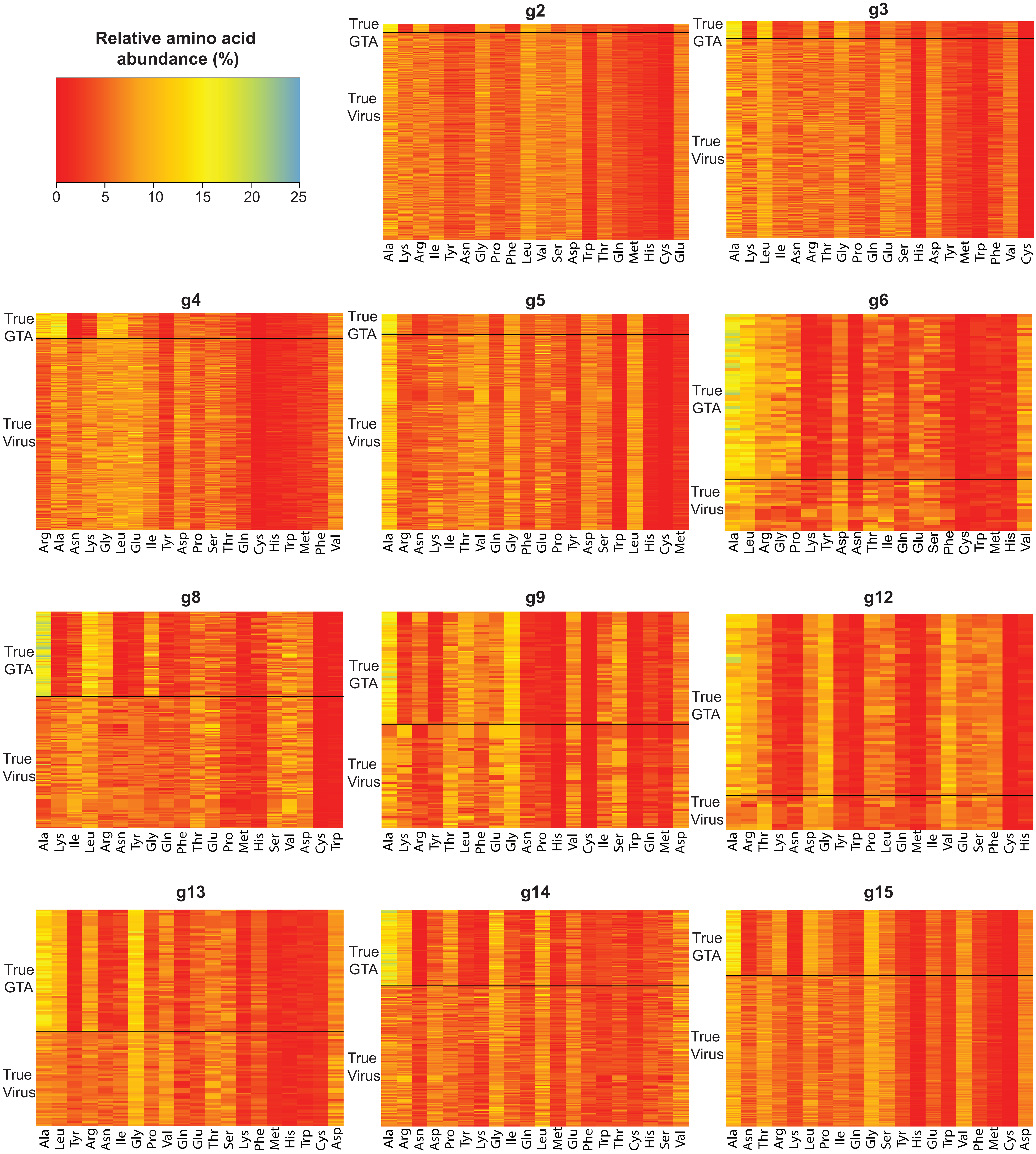

Supplementary Figure S1. The amino acid composition of viral and alphaproteobacterial homologs of the 11 RcGTA genes. These homologs were used in the training and cross-validation of the SVM classifier. Each heatmap corresponds to one of the 11 genes (see Supplementary Table S1 for the functional annotations of the genes). Each row in a heatmap corresponds to an individual homolog of the RcGTA gene. The homologs from viruses and alphaproteobacterial are separated by the black line and labeled as “True Virus” and “True GTA”, respectively. The heatmap shows the relative abundance of each amino acid within a homolog. For each gene, the amino acids on the X axis are sorted by the absolute difference in the average relative abundance between “true viruses” and “true GTAs” (from highest on the left to the lowest on the right).

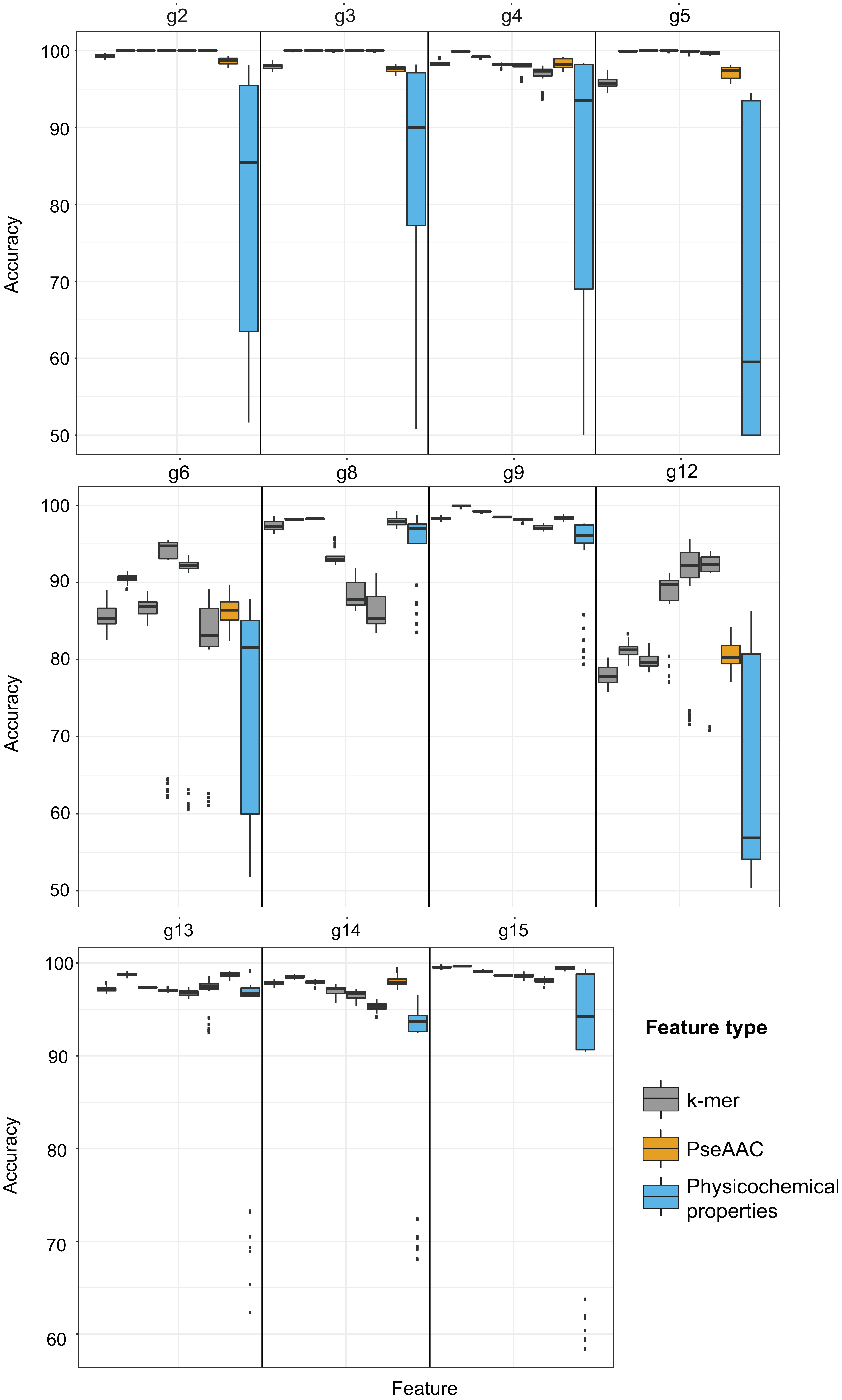

Supplementary Figure S2. The weighted accuracy scores for different types of features. The boxplots for the three feature types are color coded. The data for six examined k-mer sizes (1, 2, 3, 4, 5, 6) are shown from the left to the right on the graphs. Each boxplot shows a median value bounded by the first and third quartiles, and the whiskers depict a deviation that was calculated using the 1.5*InterQuartile Range rule. Outliers are shown as dots.
